# *In vitro *activity of telithromycin against *Haemophilus influenzae *at epithelial lining fluid concentrations

**DOI:** 10.1186/1471-2180-8-23

**Published:** 2008-01-29

**Authors:** Elena De Vecchi, Lucia Nicola, Monica Larosa, Lorenzo Drago

**Affiliations:** 1 Laboratory of Clinical Microbiology, Department of Preclinical Sciences LITA Vialba, University of Milan, Via GB Grassi 74, 20159 Milan, Italy; 2 Medical Affairs & Scientific Relations, sanofi-aventis, v.le L. Bodio 37/b – Milano Milan, Italy

## Abstract

**Background:**

*Haemophilus influenzae *is one of the main aetiological agents of community-acquired respiratory tract infections. The primary aim of this study was to evaluate the antibacterial activity of telithromycin against *H. influenzae *clinical isolates showing different pattern of resistance in comparison with azithromycin and clarithromycin at 1/4 ×, 1/2 ×, 1 ×, 2 ×, 4 × minimum inhibitory concentration (MIC) and to peak concentrations in epithelial lining fluid (ELF). The secondary aim was to determine the influence of CO_2 _enriched atmosphere on bacterial susceptibility.

**Results:**

Telithromycin showed high activity against *H. influenzae*, including strains susceptible to β-lactams (n = 200), β-lactamase producer (n = 50) and β-lactamase negative ampicillin resistant (BLNAR) (n = 10), with MIC from ≤0.03 to 4 mg/L, and MIC_50_/MIC_90 _of 1/2 mg/L with susceptibility rate of 100%, and minimum bactericidal concentrations (MBC) from 2 to 4-fold higher than the MIC. Azithromycin was the most active tested macrolide (range: 0.25 – 4 mg/L; MIC_50_/MIC_90_: 1/2 mg/L), comparable to telithromycin, while clarithromycin showed the highest MICs and MBCs (range: 0.25 – 8 mg/L; MIC_50_/MIC_90_: 2/8 mg/L). In time-kill studies, telithromycin showed a bactericidal activity at the higher concentrations (4 – 2 × MIC and ELF) against all the strains, being complete after 12 – 24 hours from drug exposition. At MIC concentrations, at ambient air, bactericidal activity of telithromycin and azithromycin was quite similar at 12 hours, and better than that of clarithromycin. Besides, telithromycin and clarithromycin at ELF concentrations were bactericidal after 12 hours of incubation for most strains, while 24 hours were needed to azithromycin to be bactericidal. Incubation in CO_2 _significantly influenced the MICs and MBCs, and only slightly the *in vitro *killing curves.

**Conclusion:**

Telithromycin showed an *in-vitro *potency against *H. influenzae *comparable to azithromycin, with an *in-vitro *killing rate more rapid and superior to clarithromycin at 2X-MIC against β-lactamase producers and BLNAR strains, and to azithromycin at ELF concentrations against β-lactamase negative strains. Against all strains, MICs and MBCs were lower in the absence of CO_2 _for the tested antibiotics, showing an adverse effect of incubation in a CO_2 _environment. The *in-vitro *potency together with the tissue concentrations of the antimicrobial, should be considered in predicting efficacy.

## Background

Telithromycin has been the first ketolide to be approved for clinical use, specifically developed for treatment of community acquired respiratory tract infections (CARTI) in order to overcome the spreading of resistance to macrolides among pneumococci [1,2]. In comparison to macrolides, telithromycin shows notable improvements in antimicrobial and pharmacokinetic properties, even if it shares with macrolides the same bacterial target, represented by the ribosome [2]. Particularly, telithromycin has significantly lower minimum inhibitory concentration (MIC) than the macrolide antibiotics for many gram-positive organisms, i.e *Streptococcus. pneumoniae *that shows, at the ribosome, a dual-site binding to the drug [3].

Telithromycin is a concentration-dependent antibiotic, thus the concentrations achieved at the infection site are recognized as an important determinant of efficacy [4]. Due to the ability to penetrate into white blood cells and being characterized by high penetration rate, it may be delivered to sites of infections and reaches elevated concentrations in several tissues, particularly in the respiratory tract, comparable to those of macrolides and superior to β-lactams [5,6]. *H. influenzae*, a major cause of CARTI [7-10], demonstrates relatively good *in vitro *susceptibility to macrolides, azalides and ketolides, which show a unimodal MIC distribution and low prevalence of high level resistance when defined by current Clinical and Laboratory Standards Institute (CLSI) breakpoints [11].

Macrolides and azalides are currently recommended for treatment of community acquired pneumonia and acute exacerbations of chronic bronchitis, with the antipneumococcal fluoroquinolones, such as levofloxacin and moxifloxacin, as alternative agents in the most severe cases [12-16].

Due to the fact that the bactericidal activity of macrolides and ketolides is related to the level of drug concentration in the infected tissue [15,16], the evaluation of antibacterial activity of concentrations achievable *in vivo*, particularly in epithelial lining fluid (ELF), against *H. influenzae*, which is one of the most common pathogen of these anatomical districts, may provide further information on telithromycin activity. Carbon dioxide has been proved to affect antibacterial activity of macrolides and ketolides, when assessed by determination of MIC values [17-20]. Influence of CO_2 _on bactericidal activity of ketolides and macrolides as measured by time kill curves has been less investigated.

The present study aimed mainly to evaluate the antibacterial activity of telithromycin concentration achievable in ELF against *H. influenzae *clinical isolates with different pattern of resistance, in comparison with azithromycin and clarithromycin and the influence of CO_2 _incubation on activity of telithromycin and macrolides.

## Methods

### Microorganisms

*H. influenzae *strains isolated from respiratory tract infections with the following phenotypes were tested: β-lactamase negative strains (n = 200), β-lactamase producer strains (n = 50), and β-lactamase negative ampicillin resistant (BLNAR) strains (n = 10). In order to avoid duplicate strains, only one isolate for each patient was considered. The strains were stored at -80°C in Haemophilus test medium (HTM) broth (Labobasi, Novazzano, CH), supplemented with 10% glycerol before testing and checked for purity throughout the study by culture and Gram staining

### Drugs

The following antibiotics, as pure substances or powder of stated potency, were considered: telithromycin (sanofi-aventis, Milan, Italy), azithromycin (Pfizer, Rome, Italy) and clarithromycin (Abbott Italy, Rome, Italy). Stock solutions of antibiotics were prepared in 95% ethanol (azithromycin) and methanol (clarithromycin and telithromycin) at concentrations of 5120 mg/L and stored in aliquots at -20°C until use. Epithelial lining fluid concentrations tested by time kill curve assay were chosen from literature data and were equal to 3.12 mg/L for azithromycin, 34 mg/L for clarithromycin and 5.4 mg/L for telithromycin [21-23]. Concentration values for all tested drugs were chosen on the basis of similar study conditions. In particular, studies on healthy volunteers were selected for this *in vitro *investigation due to the frequent inter-individual pharmacokinetics variability in patients

### Determination of minimum inhibitory concentration (MIC) and minimum bactericidal concentration (MBC)

Antibiotic susceptibilities to all the tested drugs were determined using a broth microdilution method according to the CLSI Approved Standards [24,25].

An adjusted inoculum of the tested organism was inoculated into Haemophilus test medium broth containing two fold serial dilutions of a starting antibiotic solution, so that each well contained approximately 5 × 10^5 ^cfu/mL. Results were observed after 18 h of incubation at 37°C and MIC was defined as the lowest concentration able to inhibit visible growth. Determination of MIC values were performed both in presence and in absence of 10% CO_2_. MBC was determined by plating 0.010 mL from the wells showing no visible growth on agar plates and incubating for 18–24 h in 10% CO_2 _enriched atmosphere which assures the best environment for growth of *H. influenzae*. MBC was considered as the concentration at which a 99.9% reduction in cfu occurred, when compared with the original inoculum. For each analytical series, quality controls were carried out with *H. influenzae *ATCC 49247 strains. To interpret MIC results, susceptibility breakpoints from CLSI were used: susceptible MIC ≤ 4 mg/L, ≤ 8 mg/L, ≤ 4 mg/L for azithromycin, clarithromycin and telithromycin respectively, resistant MIC > 4 mg/L, >32 mg/L and >16 mg/l for azithromycin, clarithromycin and telithromycin, respectively.

### Time kill curves

Bactericidal activity of drugs under study were evaluated by performing time kill curves experiments on all *H. influenzae *strains. HTM broth containing drug concentrations equivalent to 1/4 × MIC, 1/2 × MIC; 1 × MIC, 2 × MIC, 4 × MIC and to peak concentrations reachable by each drug in ELF was inoculated with 5 × 10^5 ^– 5 × 10^6 ^cfu/mL, and incubated at 37°C in presence or absence of 10% CO_2_. Viability counts of antibiotic containing suspensions and controls, lacking antibiotic, were obtained at 0, 3, 6 12 and 24 h by plating 10-fold dilutions of 0.1 mL aliquots from each tube onto chocolate agar plates, which were incubated for up to 48 h in CO_2 _enriched atmosphere at 37°C. A given concentration of antibiotic was considered bactericidal if it reduced the inoculum viable count by ≥ 3 log_10 _CFU/mL, or bacteriostatic if it reduced the inoculum viable count by <3 log CFU/mL

## Results

Antibacterial activity of azithromycin, clarithromycin and telithromycin against *H. influenzae *expressed as MIC and MBC values and rate of susceptibility is depicted in Tables 1 and 2, where data obtained by incubating bacteria at ambient air or at 10% CO_2 _are summarized. Telithromycin in ambient air showed activity against all the tested *H. influenzae *strains, with MIC values ranging from ≤ 0.03 to 4 mg/L, and susceptibility rates of 100%, similar to the azithromycin rates.

**Table 1:** MIC values of *H. influenzae *strains

**Microorganisms**	**Drug**	**MIC**					
		**Ambient air**			**CO_2_**		

		*Range (mg/L)*	*MIC_50_/MIC_90 _(mg/L)*	*S (%)*^#^	*Range (mg/L)*	*MIC_50_/MIC_90 _(mg/L)*	*S (%)*^#^

β-lactamase negative (n = 200)	**Tel**^§^	0.03 – 4	1/2	100	0.06 – 16	2/8	82
	**Clr**	0.25 – 8	2/8	100	1 – 16	8/16	91
	**Azm**	0.25 – 2	1/2	100	0.5 – 8	2/4	88
β-lactamase positive (n = 50)	**Tel**	0.06 – 4	1/2	100	0.125 – 8	2/8	87
	**Clr**	0.5 – 8	2/8	100	1 – 16	8/16	89
	**Azm**	0.25 – 2	1/2	100	0.5 – 8	2/4	89
BLNAR* (n = 10)	**Tel**	0.06 – 4	1/4	100	0.125 – 8	2/8	90
	**Clr**	1 – 8	1/8	100	2 – 16	8/16	90
	**Azm**	0.25 – 4	1/4	100	0.5 – 8	2/8	80

*: BLNAR: β-lactamase negative ampicillin resistant; ^§^: Tel: telithromycin; Clr: Clarithromycin; Azm: Azithromycin. ^# ^S:susceptible strains according to CLSI breakpoints: Telithromycin: susceptibility: ≤ 4 mg/L, resistance: ≥ 16 mg/L; Clarithromycin: susceptibility: ≤ 8 mg/L, resistance: ≥ 32 mg/L; Azithromycin: susceptibility: ≤ 4 mg/L.

**Table 2:** MBC values of *H. influenzae *strains

**Microorganisms**	**Drug**	**Ambient air**		**CO_2_**	
		*Range (mg/L)*	*MBC*_50_*/MBC*_90 _(mg/L)	*Range (mg/L)*	*MBC*_50_*/MBC*_90 _***(mg/L)***

β-lactamase negative (n = 200)	**Tel**^§^	0.125 – 16	1/4	0.06 – 32	4/16
	**Clr**	0.25 – 64	4/16	1 – 128	8/64
	**Azm**	0.25 – 8	1/4	0.5 – 64	4/8
β-lactamase positive (n = 50)	**Tel**	0.125 – 8	1/4	0.125 – 16	4/16
	**Clr**	0.5 – 32	4/16	1 – 64	8/64
	**Azm**	0.25 – 8	1/4	0.5 – 32	4/8
BLNAR* (n = 10)	**Tel**	0.125 – 8	1/8	0.25 – 8	4/8
	**Clr**	1 – 16	4/16	2 – 32	8/32
	**Azm**	0.25 – 8	1/8	0.5 – 16	4/16

*: BLNAR: β-lactamase negative ampicillin resistant; ^§^: Tel: telithromycin; Clr: Clarithromycin; Azm: Azithromycin.

Generally, MICs and MBCs against β-lactamase negative *H. influenzae *strains were lower in absence of CO_2 _for all the tested antibiotics, with a decrease from 2 to 4 fold in respect to the CO_2 _incubation, being MIC_50_/MIC_90 _2/8 and 1/2 mg/L for telithromycin, 8/16 and 2/8 mg/L for clarithromycin, 2/4 and 1/2 mg/L for azithromycin, after incubation with or without CO_2_, respectively.

The MBCs of telithromycin were closer to the MIC values with respect to the two comparators. MBC_50_/MBC_90 _were 4/16 and 1/4 mg/L for telithromycin, 8/64 and 4/16 mg/L for clarithromycin, 4/8 and 1/4 mg/L for azithromycin after incubation with or without CO_2_, respectively. In absence of CO_2_, all the strains were fully susceptible to the study drugs (100%). After incubation in CO_2_, the susceptibility rate was generally decreased (from 82% of telithromycin to 91% of clarithromycin), showing an interfering effect of this particular medium. The non-susceptible strains were included into the I (Intermediate) category, with the exclusion of 3 strains resistant to telithromycin. Similar results were observed for β-lactamase positive *H. influenzae *strains with MIC_50_, MIC_90_, MBC_50 _and MBC_90 _equal to those observed for β-lactamase negative strains. The microbiological activity of telithromycin was not significantly affected by β-lactamase production. Also in this case, the addition of CO_2 _to the medium influenced the microbiological results, in terms of MIC and MBC values and susceptibility rates: 100% of susceptibility in open air for all the tested drugs, 87 and 89% in CO2 respectively for telithromycin and macrolides.

The 10 BLNAR strains were fully susceptible to the antibiotics in open air medium. In CO_2 _atmosphere, MIC and MBC were higher in presence of CO_2 _for all the tested antibiotics; the activity of telithromycin and clarithromycin was slightly superior to that of azithromycin being the susceptibility rate 90%, 90% and 80%, respectively.

**Figure 1:**
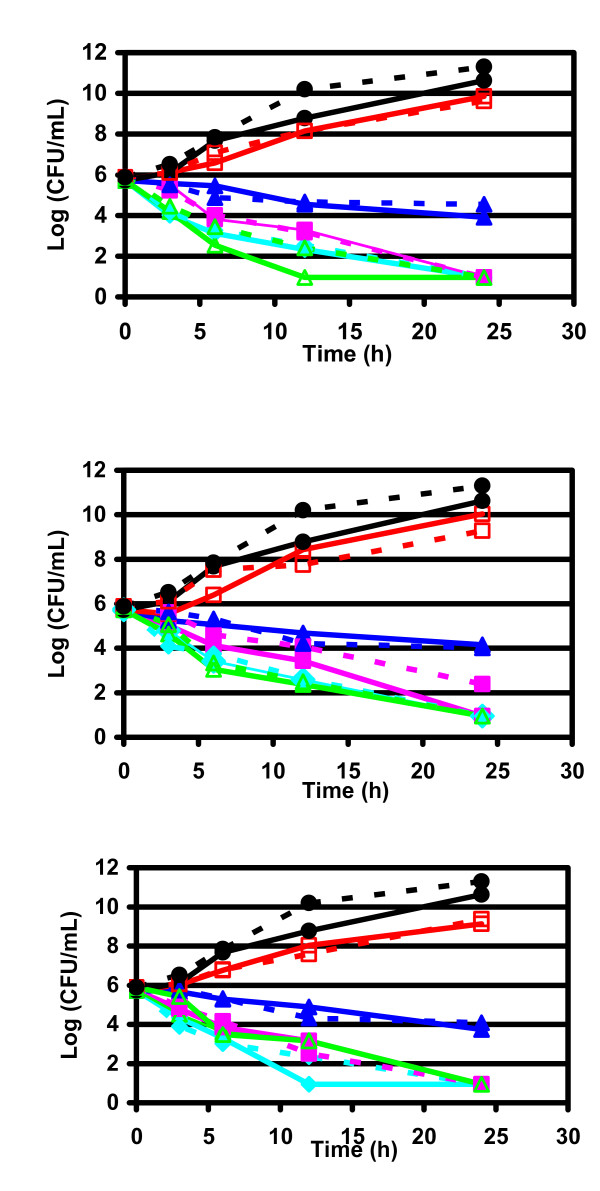
**Bactericidal activities against β-lactamase negative *H. influenzae***. Time kill curve of telithromycin (upper), clarithromycin (middle) and azithromycin (lower). black circle: Control growth (no antibiotic); red square: 1/2 × MIC; dark blue triangle: 1 × MIC; pink square: 2 × MIC; blue rhomb: 4 × MIC; green triangle: ELF. Full line: ambient air; dashed line: CO_2 _enriched atmosphere.

**Figure 2:**
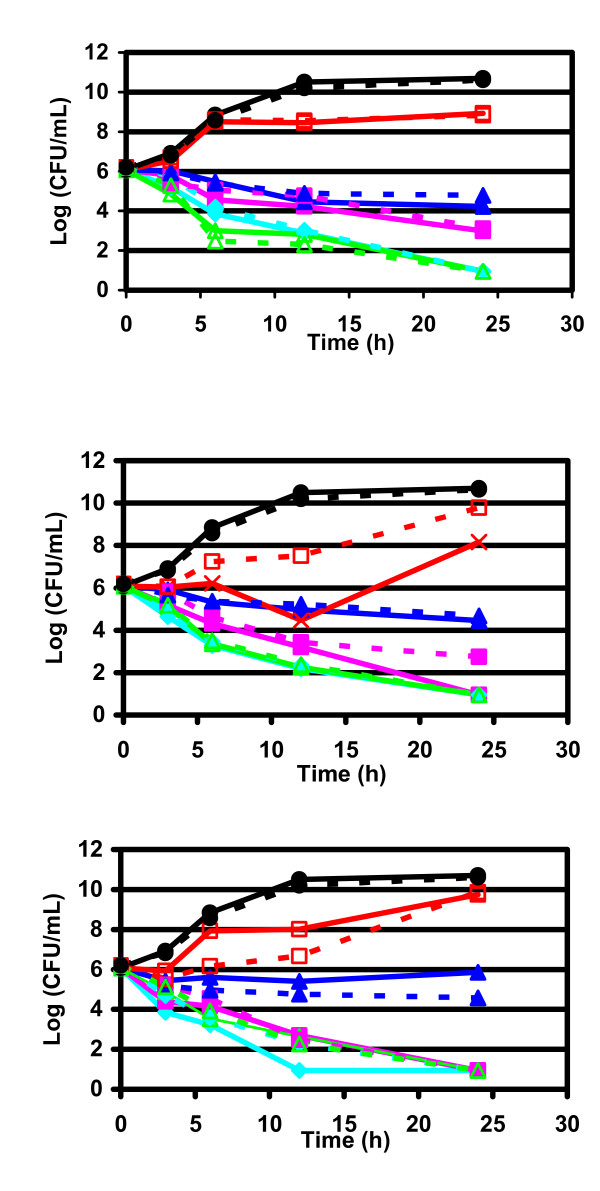
**Bactericidal activities against β-lactamase positive *H. influenzae***. Time kill curve of telithromycin (upper), clarithromycin (middle) and azithromycin (lower). black circle: Control growth (no antibiotic); red square: 1/2 × MIC; dark blue triangle: 1 × MIC; pink square: 2 × MIC; blue rhomb: 4 × MIC; green triangle: ELF. Full line: ambient air; dashed line: CO_2 _enriched atmosphere.

**Figure 3:**
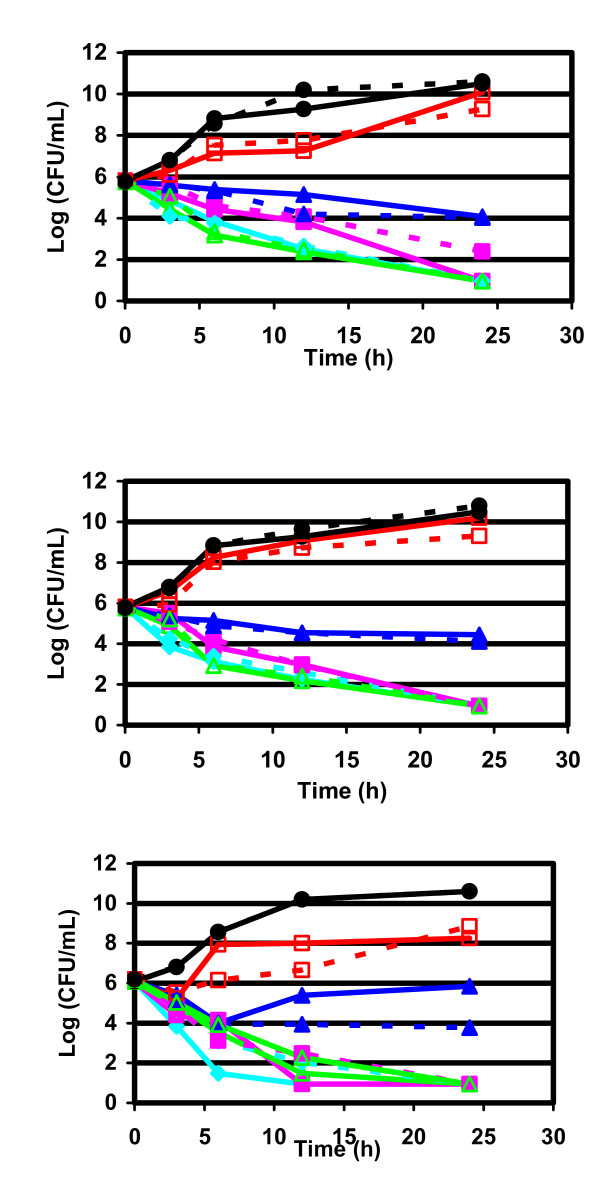
**Bactericidal activities against β-lactamase negative ampicillin resistant *H. influenzae***. Time kill curve of telithromycin (upper), clarithromycin (middle) and azithromycin (lower). black circle: Control growth (no antibiotic); red square: 1/2 × MIC; dark blue triangle: 1 × MIC; pink square: 2 × MIC; blue rhomb: 4 × MIC; green triangle: ELF. Full line: ambient air; dashed line: CO_2 _enriched atmosphere.

Results obtained in time-kill curves for azithromycin, clarithromycin and telithromycin against β-lactamase-negative, β-lactamase-positive and BLNAR *H. influenzae *are shown in Figures 1, 2, 3 and in Tables 3, 4, 5. Bactericidal activities of telithromycin, clarithromycin and azithromycin against *H. influenzae *were similar, independently from the pattern of resistance. All these drugs were fully bactericidal after 12 hours at concentration of 4 × MIC and after 24 hours at the highest concentrations (2–4 × MIC, and ELF) against β-lactamase-negative strains (Figure 1, Table 3). Telithromycin and clarithromycin were bactericidal after 12 hours of incubation also at ELF concentration, while at the same time, azithromycin was bactericidal at 2 × MIC (Figure 1).

**Table 3:** Time kill curve against β-lactamase negative *H. influenzae *strains (N = 200)

**Antibiotic**		**Mean changes in colony counts vs initial inocula after**							
		
		3 h		6 h		12 h		24 h	
		
		CO_2_	Air	CO_2_	Air	CO_2_	Air	CO_2_	Air
*Telithromycin*	4 × MIC	-1.42	-1.72	- 2.81	-2.61	> -3.0	> -3.0	> -3.0	> -3.0
	2 × MIC	-0.64	-0.18	-1.87	-1.91	-2.71	-2.45	> -3.0	> -3.0
	1 × MIC	-0.36	-0.15	-0.99	-0.26	-1.21	-1.18	-1.32	-1.81
	1/2 × MIC	0.22	0.39	1.20	0.85	2.27	2.43	3.74	4.12
	1/4 × MIC	0.43	0.35	1.51	1.35	2.94	2.54	4.93	4.59
	ELF	-1.43	-1.54	-2.43	> -3.0	> -3.0	> -3.0	> -3.0	> -3.0
*Clarithromycin*	4 × MIC	-1.03	-0.94	-2.18	-2.32	> -3.0	> -3.0	> -3.0	> -3.0
	2 × MIC	-0.75	-0.69	-1.80	-1.61	-2.42	-2.30	> -3.0	> -3.0
	1 × MIC	-0.23	-0.49	-0.75	0.66	-0.91	-1.04	-1.18	-1.58
	1/2 × MIC	-0.19	-0.19	0.69	0.66	1.57	2.71	3.81	4.31
	1/4 × MIC	0.51	0.45	1.69	1.40	2.93	2.99	5.01	4.84
	ELF	-1.09	-1.26	-2.68	> -3.0	> -3.0	> -3.0	> -3.0	> -3.0
*Azithromycin*	4 × MIC	-1.99	-1.38	-2.86	-2.44	> -3.0	> -3.0	> -3.0	> -3.0
	2 × MIC	-0.98	-0.94	-1.73	-1.88	> -3.0	-2.57	> -3.0	> -3.0
	1 × MIC	-0.10	-0.06	-0.56	-0.45	-1.55	-1.84	-1.80	-1.99
	1/2 × MIC	0.18	0.29	0.91	1.03	1.71	2.31	3.49	3.41
	1/4 × MIC	0.39	0.36	1.38	1.63	2.23	2.53	4.58	4.28
	ELF	-0.45	-1.22	-2.40	-2.12	-2.72	-2.55	> -3.0	> -3.0
*Control*		0.64	0.38	1.96	1.95	4.32	3.05	5.44	4.90

**Table 4:** Time kill curve against β-lactamase positive *H. influenzae *(n = 50)

**Antibiotic**		**Changes in colony counts vs initial inocula after**							
		
		3 h		6 h		12 h		24 h	
		
		CO_2_	Air	CO_2_	Air	CO_2_	Air	CO_2_	Air
*Telithromycin*	4 × MIC	-1.28	-1.44	-2.74	-2.80	> -3.0	> -3.0	> -3.0	> -3.0
	2 × MIC	-0.37	-0.89	-1.57	-1.77	-2.77	-2.88	> -3.0	> -3.0
	1 × MIC	-0.53	-0.16	-0.82	-0.76	-0.96	-1.12	-1.51	-1.62
	1/2 × MIC	-0.13	-0.04	1.06	0.13	1.33	1.61	3.59	2.08
	1/4 × MIC	0.33	0.41	1.66	1.29	2.46	2.74	4.03	3.19
	ELF	-1.00	-0.96	-2.68	-2.72	> -3.0	> -3.0	> -3.0	> -3.0
*Clarithromycin*	4 × MIC	-1.47	-0.88	-2.00	-2.22	> -3.0	> -3.0	> -3.0	> -3.0
	2 × MIC	-0.80	-0.30	-1.14	-1.51	-1.42	-1.86	> -3.0	> -3.0
	1 × MIC	-0.27	-0.06	-0.79	-0.60	-1.31	-1.61	-1.40	-1.86
	1/2 × MIC	0.52	0.45	2.43	2.42	2.36	2.38	2.63	2.84
	1/4 × MIC	0.71	0.69	2.69	2.47	2.73	3.25	3.91	3.98
	ELF	-0.91	-1.22	-2.65	> -3.0	> -3.0	> -3.0	> -3.0	> -3.0
*Azithromycin*	4 × MIC	-1.49	-2.22	-2.61	-2.85	> -3.0	> -3.0	> -3.0	> -3.0
	2 × MIC	-0.98	-1.68	-1.65	-1.93	> -3.0	> -3.0	> -3.0	> -3.0
	1 × MIC	-1.03	-0.64	-1.23	-0.48	-1.43	-0.68	-1.62	-0.23
	1/2 × MIC	-0.67	-0.15	-0.03	1.86	0.47	1.93	3.67	2.18
	1/4 × MIC	0.29	0.26	1.58	1.96	2.34	2.13	4.12	3.28
	ELF	-1.05	-1.20	-2.27	-2.53	> -3.0	> -3.0	> -3.0	> -3.0
*Control*		0.62	0.73	2.38	2.49	4.02	4.13	4.44	4.55

**Table 5:** Time kill curve against β-lactamase negative-ampicillin resistant *H. influenzae *(n = 10)

**Antibiotic**		**Changes in colony counts vs initial inocula after**							
		
		3 h		6 h		12 h		24 h	
		
		CO_2_	Air	CO_2_	Air	CO_2_	Air	CO_2_	Air
*Telithromycin*	4 × MIC	-1.53	-1.96	-2.38	-2.69	> -3.0	> -3.0	> -3.0	> -3.0
	2 × MIC	-0.68	-0.30	-1.55	-1.96	-2.82	-2.84	> -3.0	> -3.0
	1 × MIC	-0.38	-0.56	-0.88	-0.66	-1.23	-1.27	-1.66	-1.37
	1/2 × MIC	0.17	0.78	2.26	2.43	2.94	3.26	3.53	4.40
	1/4 × MIC	0.33	0.92	2.66	2.68	3.26	3.30	3.64	4.51
	ELF	-0.55	-0.92	-2.85	-2.89	> -3.0	> -3.0	> -3.0	> -3.0
*Clarithromycin*	4 × MIC	-1.68	-0.96	-2.02	-1.96	> -3.0	> -3.0	> -3.0	> -3.0
	2 × MIC	-0.16	-0.62	-1.16	-1.38	-1.70	-1.99	> -3.0	> -3.0
	1 × MIC	-0.14	-0.21	-0.45	-0.42	-1.56	-0.67	-1.75	-1.74
	1/2 × MIC	0.33	0.51	1.76	1.34	1.99	1.45	3.51	4.28
	1/4 × MIC	0.64	0.75	2.87	2.88	3.44	3.35	4.03	4.61
	ELF	-0.71	-1.36	-2.43	-2.62	> -3.0	> -3.0	> -3.0	> -3.0
*Azithromycin*	4 × MIC	-1.55	-1.62	-2.50	-2.76	> -3.0	> -3.0	> -3.0	> -3.0
	2 × MIC	-1.16	-1.37	-1.90	-1.96	> -3.0	> -3.0	> -3.0	> -3.0
	1 × MIC	-1.01	-0.56	-0.90	-0.89	-1.68	-1.06	-1.78	-1.59
	1/2 × MIC	0.21	0.72	0.92	1.28	1.98	2.01	2.49	2.53
	1/4 × MIC	0.27	0.79	2.54	2.04	3.04	3.14	3.72	4.16
	ELF	-1.12	-1.40	-2.57	-2.73	-2.83	> -3.0	> -3.0	> -3.0
*Control*		0.97	0.98	3.07	3.01	3.87	3.46	5.05	4.68

Similar trends in bactericidal activities were observed for β-lactamase producer and BLNAR strains (Figures 2 and 3, Tables 4 and 5).

Incubation of the bacterial culture in open air or in CO_2 _seemed to slightly influence the killing curves, as, globally, no marked differences in bactericidal activity were observed between incubation in the presence or absence of CO_2_.

## Discussion

The *in vitro *activity of telithromycin, the first ketolide developed for clinical use, has been widely evaluated in international and local studies, demonstrating a spectrum of activity that encompasses the key respiratory pathogens, including *H. influenzae*. As the bactericidal activity of macrolides and ketolides is related to the magnitude of drug concentration at the site of infection, it is instrumental the evaluation of antimicrobial activity of concentrations achievable *in vivo *at the bronchial tree. Thus the present study assessed the comparative *in vitro *bacteriological activity and the killing kinetics of telithromycin, azithromycin and clarithromycin against *H. influenzae *at concentrations multiple of the MIC and equal to ELF.

Telithromycin in ambient air showed activity against all the tested *H. influenzae *strains, with susceptibility rates of 100%, similar to azithromycin. The respective MBCs were from 2 to 4 fold higher than the MIC, generally lower than the comparators. Previous *in vitro *studies have already showed that the *in vitro *potency against *H. influenzae *of telithomycin is similar to azithromycin, considered the most active macrolide against this pathogen, and superior to clarithomycin [7,11,26-33].

The incubation in carbon dioxide affected the antibacterial activity of all the tested antibiotics, causing a notable increase in MICs and MBCs, resulting in a decreased rate of susceptibility among *H. influenzae *strains.

Susceptibility testing of respiratory tract pathogens is often performed in a CO_2 _environment to ensure that the bacteria grow faster; however in this ambient the pH of the test medium decrease and macrolides and telithromycin activity seems adversely affected by this pH decrease. Thus the results of our study show that telithromycin susceptibility should be tested in ambient air, as well as that of macrolides. Notably, other recent *in vitro *studies have highlighted the adverse impact of CO_2 _on susceptibility testing of telithromycin in key respiratory pathogens including *H. influenzae *[17-19].

Few studies have evaluated the bactericidal activity of telithromycin alone or in comparison with macrolides against respiratory pathogens, and in particular against *H. influenzae *[8,32,33]. Our data confirm the results of these studies, indicating that the bactericidal activity of telithromycin is mainly evident at concentrations as high as twice and four times the MIC. For all the tested antibiotics the killing of *H. influenzae *was not affected by different resistance patterns of the strains included into the study. When MIC concentrations were considered, bactericidal activities of the studied drugs were quite similar, with azithromycin showing a more rapid killing at 2 × MIC. However, although not fully bactericidal, activity of telithromycin against *H. influenzae *seemed superior in comparison with that of clarithromycin and close to that of azithromycin. By contrast, when bactericidal activity of pulmonary concentrations was tested, telithromycin and clarithromycin showed a higher rate of killing than azithromycin on some strains, probably due to the inferior tissue distribution of this drug.

As both telithromycin and azithromycin are concentration dependent antibiotics, their penetration rate in site infection is an important determinant in predicting efficacy, thus MIC, breakpoints of macrolides, azalides and ketolides against this organism must be considered together with their levels in respiratory tissues and ELF. There is growing evidence that, even though MICs for macrolides against *H. influenzae *may be in the 'susceptible' range (as defined by current MIC breakpoints), *in vivo *bacteriological efficacy is poor against this pathogen, while PK/PD derived breakpoints seems to be more consistent with clinical outcomes [34]. For both clarithromycin and azithromycin, the PK/PD breakpoint is five doubling dilutions lower than the CLSI breakpoint [35], while for telithromycin a breakpoint of 0.5 mg/L has been proposed [36]. By considering these values, all the strains evaluated in the present study should be considered resistant to the two macrolides, while some of them should be classified as susceptible to telithromycin. However, determination of PK/PD breakpoints is usually based on serum concentrations chosen for optimal bacterial eradication and may not reflect the actual concentration at the site of infection, as occurs for the tested drugs which provide higher concentrations in the lungs than in other compartments, thus allowing higher susceptibility breakpoints when treating pulmonary infections.

## Conclusion

In conclusion, telithromycin showed an *in-vitro *potency against *H. influenzae *comparable to azithromycin, with a superior *in-vitro *killing rate to clarithromycin at 2X-MIC against β-lactamase producers and BLNAR strains, and to azithromycin at ELF concentrations against β-lactamase negative strains. Against all strains, MICs and MBCs were lower in the absence of CO_2 _for the tested antibiotics, showing an adverse effect of incubation in a CO_2 _environment. The *in-vitro *potency together with the pharmacokinetic profile of the antimicrobial, should be considered in predicting its efficacy in the empirical therapy of CARTI.

## Competing interests

LD received research funding from sanofi aventis.

## Authors' contributions

LD participated in design and coordination of the study, interpretation of the data, and co-drafted the manuscript. EDV participated in study design, co-performed killing curves, analysis and interpretation of the data and co-drafted the manuscript. LN co-performed time kill curves and participated in analysis and interpretation of the data. ML participated in revising the manuscript.

All authors read and approved the final manuscript.
